# Special Issue “Advances in Retinal Diseases: 2nd Edition”

**DOI:** 10.3390/ijms27052187

**Published:** 2026-02-26

**Authors:** Minzhong Yu

**Affiliations:** 1 Department of Ophthalmology and Visual Sciences, University Hospitals Eye Institute, Case Western Reserve University, 11100 Euclid Ave., Cleveland, OH 44106, USA; minzhong.yu@case.edu; 2 Cole Eye Institute, Cleveland Clinic Foundation, 2022 E 105th St., Cleveland, OH 44106, USA; 3 Department of Ophthalmology, Cleveland Clinic Lerner College of Medicine, Case Western Reserve University, 9501 Euclid Ave., Cleveland, OH 44195, USA

Retinal diseases continue to command the attention of clinicians and scientists alike because they compress complex biology into the span of a single, fragile tissue and translate molecular perturbations into life-altering vision loss. In the last decade, the field has moved beyond single-pathway explanations toward adopting a network view in which metabolism, inflammation, gene regulation, vascular biology, and structural integrity interact bidirectionally. This second edition of the Special Issue “Advances in Retinal Diseases” assembles fourteen contributions that achieve more than adding data points; together, they clarify mechanistic relationships, challenge comfortable assumptions, and indicate pragmatic routes for translating discoveries into lasting benefits for patients. What follows is a synthesis of these lessons—not an inventory of results but a narrative about where retinal science is going and why the work contained herein will help it get there. Beyond molecular and imaging advances, visual electrophysiology (ERG, EOG, and VEP) remains indispensable for translating gene-level variation into clinically actionable functional phenotypes in systemic hereditary syndromes.

A recurring motif across this Special Issue is metabolic precision. The retina’s energy budget is famously unforgiving; minor inefficiencies can tip a delicate balance toward dysfunction. In neovascular disease, the insight that endothelial cells are not merely passengers of angiogenic growth factors but active metabolic decision-makers reframes how we think about treatment. By detailing how glycolysis, lipid handling, and mitochondrial signaling shape endothelial phenotypes, investigators have shown that vessel growth, leakage, and even responsiveness to therapy are constrained by the metabolic programming of the endothelium itself. This perspective dovetails with broader evidence of photoreceptor and retinal pigment epithelium (RPE) vulnerability when substrate delivery, mitochondrial quality control, or antioxidant capacity fall short. Metabolism thus emerges not as background physiology but as a set of levers—glycolytic flux, fatty-acid oxidation, and redox balance—that can be tuned to normalize aberrant angiogenesis or stabilize stressed neurons and supporting cells.

Equally persistent is the theme of inflammation as both a driver and an amplifier of retinal injuries. Single-cell and pathway-level analyses across models of diabetic retinopathy, uveitis, ischemia-reperfusion, and inherited degeneration reveal microglia and infiltrating myeloid cells that are neither uniformly damaging nor uniformly protective. Instead, their effect depends on timing, metabolic state, and local cues emitted by Müller glia, neurons, and endothelium. Complement activation, cytokine signaling, and oxidative bursts intersect with metabolic stress to produce self-reinforcing cycles of dysfunction—cycles that become increasingly resistant to late intervention. The translational implication is that we must treat immune modulation as a phase-specific task, requiring a solution that can be implemented early enough to prevent irreversible synaptic and structural loss, that is targeted enough to preserve reparative microglial programs, and that is durable enough to accommodate the relapsing courses of many retinal diseases.

The gene-therapy arc represented in this Special Issue is notable for its breadth. On one end are isoform-aware frameworks for inherited retinal diseases that move beyond locus-level thinking to ask which splice variants matter, when, and in which cells. This granularity is not cosmetic; vector payloads, promoter choices, and outcome measures all hinge on it. On the other end are gene-based strategies aimed at complex disease, where the goal is not a ‘cure’ but persistent expression of protective proteins—complement modulators, neurotrophic factors, or metabolic buffers—that reduce treatment burdens while addressing mechanisms upstream of atrophy and circuit failure. Between these poles sit concrete steps forward: functional in vitro assays that validate activity before animal work; clever use of alternative electron-transport enzymes to support neurons under bioenergetic strain; and platform-level attention to biodistribution, immune quietude, and durability. The cumulative message is that the field’s maturing toolkit is best applied when guided by isoform-level biology and a sober appreciation of what durability should mean in diseases that unfold over years.

Diagnostic genetics has also earned a critical reappraisal. Whole-genome sequencing is often imagined as a universally superior successor to exome panels. The reality is more nuanced: the added diagnostic yield depends on the fidelity of copy-number and structural variant detection, pipelines that can recognize and prioritize deep-intronic splice changes, and—crucially—a feedback loop between the lab and the clinic. Reanalysis of earlier exomes with improved algorithms and updated gene–disease relationships can rescue a surprising fraction of previously negative cases. At the same time, deep-intronic and regulatory variants, when present, rarely speak for themselves; they demand functional corroboration. The pragmatic conclusion is not to romanticize technology tiers but to invest in interpretation: robust CNV calling, judicious use of genome sequencing when it can change management, and routine re-interrogation as knowledge evolves.

Neurovascular disease—especially age-related macular degeneration (AMD)—remains an instructive proving ground for this synthesis of mechanisms and translation. The contributions in this volume sharpen a panoramic view of dry AMD in which oxidative injury, complement dysregulation, mitochondrial impairment, ferroptosis susceptibility, impaired autophagy, and lipid traffic at the RPE–Bruch’s membrane interface collaborate to produce drusen and, eventually, atrophy. This multi-node network has therapeutic consequences. Complement inhibition alone cannot substitute for neuronal and synaptic protection; metabolic support without inflammation control is likely to stall, as underscored by recent GA trials that achieved regulatory milestones but leave room for mechanism-guided combinations [1,2].

One particularly timely thread is the reframing of endothelial metabolism as a target in neovascular AMD. By showing how glycolytic flux and lactate signaling gate endothelial sprouting, junctional stability, and paracrine crosstalk with macrophages, these studies turn concepts from tumor angiogenesis toward the eye without importing unhelpful baggage. The result is a specific, testable idea: partial, transient down-tuning of glycolysis can normalize pathological angiogenesis without collapsing the physiological vasculature. When layered with anti-VEGF therapy or delivered via long-acting platforms, metabolic modulation may improve durability and rescue patients whose disease has become refractory to single-axis blockades.

A complementary perspective comes from the field of neonatal disease. In retinopathy of prematurity, pathologic angiogenesis emerges from a sequence that couples hyperoxia-driven vaso-obliteration with hypoxia-driven neovascularization, with microglia, astrocytes, and Müller cells serving as both amplifiers and moderators. Natural-product pharmacology—long a source of therapies for cardiometabolic and infectious diseases—receives a clear-eyed assessment here. The point is not that ‘natural’ equals ‘benign’ or ‘effective’ but that rigorous chemistry, mechanism-of-action studies, and careful model selection can surface small molecules with anti-angiogenic, antioxidant, or anti-inflammatory activity suited to the unique safety calculus of premature infants. Coupled with advances in materials and ocular delivery, such agents may one day complement laser and antibody therapies by smoothing the peaks of disease activity rather than chasing them.

Infectious retinopathy often sits at the edge of such discussions, yet it offers an instructive counterexample: in this setting, metabolic tweaking alone is insufficient because virions exploit host metabolism in ways that outpace incremental correction. Research examining entry-stage blockade and adsorption interference in retinal cells is provocative for two reasons. First, it emphasizes the value of looking beyond replication to earlier stages where small molecules can meaningfully lower inocula by blocking receptor engagement or destabilizing the viral envelope. Second, it models a translational workflow—chemical profiling, cytotoxicity and antiviral assays, and docking to the virus–host interface—that can be ported to other ocular pathogens. In a landscape where standard antivirals struggle with resistance and bioavailability, such a scaffold for discovery is welcome. Acute presentations also remind us that the retina can be the canary in the systemic coal mine. A case in which floaters heralded a myeloproliferative disorder underscores the importance of a systemic work-up when hemorrhagic signatures defy local explanations, especially in younger patients.

Imaging and analytics form the connective tissue that binds mechanisms to clinics. Adaptive optics, OCT, and OCT-angiography have evolved from documentation tools into hypothesis engines, enabling earlier and more objective staging in uveitis and AMD. By correlating ellipsoid-zone integrity, outer-segment reflectivity, microvascular density, and dark-adaptation dynamics with proteomic and transcriptomic states, these methods allow us to stage disease earlier and more objectively. This staging then feeds back into model selection, powering trials that are enriching for the patients most likely to benefit from a given mechanism and providing sensitive endpoints for therapies designed to slow rather than reverse structural change.

Delivery science threads through many advances. Long-acting depots and refillable reservoirs promise to trade pharmacokinetic peaks and troughs for steady exposure; suprachoroidal access and improved formulations reduce anterior-segment spillover; vector engineering pursues cellular specificity with immune quietude. Yet durability without control is not an asset. The most attractive platforms are those that incorporate titration, cessation, and rescue, because patient trajectories are not monotonic and safety in a tissue as unforgiving as the eye depends on reversible decisions. The pragmatic bar is high: fewer visits, stable structure and function, easy monitoring, and a safety margin generous enough to withstand the surprises that accompany first-in-class interventions. Long-acting systems that enable durable delivery with clinic-friendly maintenance, such as refillable reservoirs, illustrate both the promise and the design trade-offs [3,4].

Taken together, the papers in this Special Issue suggest making four practical commitments for the next stage of retinal therapeutics. First, lean into mechanism-guided combinations: pair complement modulation with neuroprotection in atrophy, metabolic support with anti-VEGF in neovascular disease, and immune calibration with synaptic preservation in inflammatory injuries. Second, insist on phase-appropriate endpoints and staging: measure what matters most at each step of the disease arc rather than waiting for visual acuity to change. Third, let models serve the question: use organoids and human tissue to rank targets and identify liabilities, rodent models to test direction of effect and dose, and non-human primates when macular fidelity is non-negotiable. Fourth, design for durability with exit ramps, not just on-ramps: long-acting therapies should anticipate success and failure with equal humility.

Finally, it is worth reflecting on what this collection reveals about the scientific culture within our field. The strongest contributions are collaborative not merely in terms of authorship but at the level of ideas. Geneticists engage with imaging scientists. Cell biologists consider clinical trial design. Pharmacologists account for patient burden. Data scientists anchor their models in pathophysiological plausibility. This interdisciplinarity is not decorative. Retinal disease does not yield to single-discipline solutions. If the advances captured in these pages are to mature into therapies that meaningfully alter patients’ lives, it will be because we continue to ask mechanistic questions with clinical intent and pursue translation with the same rigor that guides discovery.

The retina demands precision and exposes complacency. The studies gathered in this Special Issue move the field toward a future defined by sharper biological definition, with greater clarity regarding cell type, disease stage, mechanism, and clinically meaningful outcomes. At the same time, they challenge us to move beyond incremental progress that leaves patients burdened by frequent visits, partial rescues, or unresolved safety concerns. If this collection achieves anything beyond presenting new data, let it help recalibrate our collective focus toward earlier detection, mechanism-aligned intervention, and durable benefits that meaningfully improve how patients see and live.

## Data Availability

Data is contained within the article.
